# Prevalence of iron deficiency in patients admitted to a geriatric unit: a multicenter cross-sectional study

**DOI:** 10.1186/s12877-024-04719-6

**Published:** 2024-01-30

**Authors:** Bertrand Fougère, François Puisieux, Pascal Chevalet, Cédric Annweiler, Emeline Michel, Laure Joly, Frédéric Blanc, Abdelghani EL Azouzi, Valérie Desré-Follet, Patrice Cacoub, on behalf of the CARENFER PA study group*, Anne-Sophie Billet, Florent Fiasson, Antoine Garnier-Crussard, Isabelle Goidin, Marc Paccalin, Laure Parnet, Mikel Sarasibar, Caroline Thomas

**Affiliations:** 1 Division of Geriatric Medicine, Tours University Hospital, Tours, France; 2 Lille Université, CHU Lille, Pôle de Gérontologie, Lille, France; 3 Nantes Université, CHU Nantes, Pôle de Gérontologie Clinique, Nantes, France; 4 Department of Geriatric Medicine and Memory Clinic, Research Center On Autonomy and Longevity, University Hospital & Laboratoire de Psychologie Des Pays de La Loire, LPPL EA 4638, SFR Confluences, University of Angers, Angers, France; 5 Université Côte d’Azur, Centre Hospitalier Universitaire de Nice, Nice, France; 6 Université Côte d’Azur, LAHMESS, Nice, France; 7 Geriatric Department, CHRU Nancy, Université de Lorraine, Nancy, France; 8 INSERM, DCAC, Université de Lorraine, Vandœuvre-Lès-Nancy, France; 9 Team IMIS, ICube Laboratory, UMR 7357 and FMTS (Fédération de Médecine Translationnelle de Strasbourg), University of Strasbourg and CNRS, Strasbourg, France; 10 3CM2R (Research and Resources Memory Centre), Geriatrics Department, Day Hospital and Cognitive-Behavioral Unit University Hospitals of Strasbourg, Strasbourg, France; 11 Geriatric Department, Dunkirk, CH Dunkirk France; 12 CSL Vifor, Paris La Défense, France; 13 UMR 7211, and Inflammation-Immunopathology-Biotherapy Department (DHU i2B), Sorbonne Universités, UPMC Univ Paris 06, Paris, France; 14 Department of Internal Medicine and Clinical Immunology, AP-HP, Groupe Hospitalier Pitié-Salpêtrière, Paris, France; 15 CHRU Tours – Pôle Vieillissement, Hôpital Bretonneau, 2 Boulevard Tonnelé, 37044 Tours Cedex 9, France

**Keywords:** Iron deficiency, Older patient, Anemia, Serum ferritin, Intravenous iron, Transferrin saturation

## Abstract

**Background:**

Iron deficiency (ID) is often associated with other comorbidities in older patients and is a factor of morbimortality. However, the prevalence of ID remains poorly documented in this population.

**Methods:**

The CARENFER PA study was a French multicenter cross-sectional study whose objective was to evaluate ID in patients (> 75 years) admitted to a geriatric unit. The primary endpoint was the ID prevalence defined as: serum ferritin < 100 µg/L and/or transferrin saturation coefficient (TSAT) < 20%. The Short Physical Performance Battery (SPPB) test was used to identify older patients at high risk of adverse events (e.g., disability, falls, hospitalization, death).

**Results:**

A total of 888 patients (mean age, 85.2 years; women, 63.5%) from 16 French centers were included from October 2022 to December 2022. The prevalence of ID was 57.6% (95% CI, 54.3–60.9) in the cohort of older patients (62.6% in anemic and 53.3% in non-anemic patients; *p* = 0.0062). ID prevalence increased significantly with the presence of more than three comorbidities (65.6% vs. 55.9%; *p* = 0.0274), CRP ≥ 12 mg/L (73.0% vs. 49.3%; *p* < 0.001) and treatment that may influence ID/anemia (60.5% vs. 49.6%; *p* = 0.0042). In multivariate analysis, only CRP ≥ 12 mg/L was an independent predictive factor of ID (odds ratio, 2.78; 95% CI, 1.92–4.08; *p* < 0.001). SPPB scores were low (0–6) in 60.5% of patients with ID versus 48.6% of patients without ID (*p* = 0.0076).

**Conclusion:**

More than half of older patients had ID, including non-anemic patients. ID was associated with the presence of inflammation and a low SPPB score.

**Trial registration:**

NCT05514951.

**Supplementary Information:**

The online version contains supplementary material available at 10.1186/s12877-024-04719-6.

## Introduction

In addition to its key role in the synthesis of hemoglobin (Hb) and oxygen transport, iron is an essential element in many physiological processes such as energy metabolism, cardiac and peripheral muscle function [1]. Therefore, even though iron deficiency (ID) is a common cause of anemia, it can also be responsible for physiological and clinical disorders before the onset of anemia [2]. Anemia is the most common hematological disorder encountered in geriatric practice. Its prevalence, in the general ambulatory population, has been evaluated between 10 and 15% after 65 years of age and more than 20% beyond 85 years of age [3]. Approximately one third of cases of anemia in the older age group are attributable to iron, folic acid or B12 deficiency [3]. Anemia is associated with increased morbidity in terms of cardiac complications, cognitive decline, frailty, hospitalizations, impaired quality of life and increased mortality [4, 5].

ID is common in chronic diseases such as heart failure, chronic kidney disease (CKD) or cancer [6–9]. Independently of anemia, ID is also associated to more rapid clinical worsening in heart failure [10], non-dialysis CKD [11, 12] and cancer [13] and is a risk factor of mortality in patients with heart failure [10, 14] and CKD [11, 12]. Indeed, the treatment of ID allows restoring Hb levels, reduces the prescription of erythropoiesis-stimulating agents (ESA) and the need for transfusion in oncology [15], gastroenterology [16, 17] and nephrology [18–22]. The most useful biomarkers for assessing iron status are serum ferritin, which reflects iron stores, and transferrin saturation (TSAT), which is indicative of the iron transported in the circulation and available for cell metabolism [23]. ID is absolute when iron reserves are low; it is functional when iron mobilization is impaired despite normal reserves (and therefore not available for hematopoiesis), generally due to chronic inflammation which blocks intestinal iron absorption and inhibits iron export from macrophage and hepatocyte iron stores [2, 24].

The frailty of older patients, who often have several comorbidities, indicates the importance of better diagnosing ID in this population. However, there is no consensus definition of ID in the older population and no specific thresholds for biomarkers of iron metabolism. Nevertheless, taking into account recent publications on ID in different conditions (heart failure, chronic kidney disease, cancer) which are frequent in the older population, there is an emerging consensus to define ID in these chronic diseases by serum ferritin < 100 μg/L and/or TSAT < 20% [25–27]. Using this definition of ID, we evaluated the prevalence of ID in a national multicenter study in older patients admitted to a geriatric unit (hospitalized or outpatients); we also assessed the association between ID and a low SPPB score, which is a marker of a high risk of adverse events (e.g., disability, falls, hospitalization, death).

## Methods

### Type of study and patients

The CARENFER PA (“*CARence EN FER Personnes Âgées*”) study was a French multicenter cross-sectional study performed in geriatric centers. The study was interventional only because blood sampling and SPPB test were done at inclusion.

The main objective of this study was to evaluate the prevalence of ID in patients 75 years of age or older admitted to a geriatric unit. A secondary objective was to evaluate the association between ID and SPPB test score.

Patients were consecutively included if they met the following criteria: patient aged over 75; patient hospitalized in a geriatric ward or seen on an outpatient basis; patient with written consent; patient affiliated with or benefiting from a social security insurance. Protected patients were excluded (i.e. subject to a legal protection measure: guardianship, curatorship or safeguard of justice). Patients unable to understand what was asked of them were not included.

The consent was explained to the patient by the geriatrician. If the physician suspected cognitive decline, consent was explained to the patient's relative in the patient's presence. If the geriatrician thought the patient had not understood and/or the patient's relative objected to the consent, the patient was not included in the study.

The study conformed to the principles of the Declaration of Helsinki and Good Clinical Practice Guidelines. It was approved by a national independent Ethics Committee (“Comité de Protection des Personnes Ile de France III”) and written informed consent was obtained from all subjects participating in the trial. The trial was registered prior to patient enrollment at clinicaltrials.gov (NCT05514951, https://www.clinicaltrials.gov/ct2/show/NCT05514951; Date of registration: August 25, 2022).

### Data collected

The main data collected during the inclusion visit were the followings: demographics (age, gender, height, weight), admission to geriatric unit (hospitalized, outpatient), comorbidities, treatment of anemia and/or iron deficiency (oral and intravenous iron, ESA, transfusion) and concomitant treatments possibly influencing ID or anemia (anticoagulant, proton pump inhibitor, platelet antiaggregant, other).

A blood test was performed within 48 h after inclusion for serum electrolytes, serum ferritin, TSAT, Hb, C-reactive protein (CRP), albumin and serum creatinine.

The Short Physical Performance Battery (SPPB) test was used to identify older patients at high risk of adverse events (e.g., disability, falls, hospitalization, death). It is composed of 3 parts: balance, repeated chair stands and gait speed [28]. Each of the three domains was scored from 0 (worst) to 4 (best) with a total score from 0 to 12 [29]. The global SPPB score interpretation was the following: 0–6, poor performance; 7–9, intermediate performance; 10–12, high performance.

### Statistical analyses

The primary endpoint was the prevalence of ID defined as serum ferritin < 100 µg/L and/or TSAT < 20% [26]. Absolute ID was defined as serum ferritin < 100 µg/L and functional ID as serum ferritin ≥ 100 µg/L and TSAT < 20%.

ID prevalence was described with the Agresti-Coull confidence interval (CI). The analysis population included all patients who met the eligibility criteria and completed a blood test within 2 days of signing the written consent.

In the literature, ID prevalence in patients over 65 years of age is 10–15% [3]. With an assumption of 15% for the prevalence of ID in older patients, a precision of 2.4%, a first-order risk of 5% and 5% of patients not evaluable, 900 patients were to be included in this study.

Anemia was defined as Hb < 13 g/dL for men and Hb < 12 g/dL for women, according to World Health Organization (WHO) [30].

A multivariate logistic regression analysis was performed to define the factors associated to ID. Variables that were statistically significant in univariate analysis at a threshold ≤ 0.2 were entered into a multivariate analysis to be tested in a multivariate model with a risk alpha level set at *p* = 0.05.

The statistical analysis was performed with SAS software version 9.4 (SAS Institute, Inc., Cary, North Carolina, USA).

## Results

### Patient disposition and characteristics

A total of 902 patients were included in 16 centers from October 5, 2022 to December 9, 2022; 14 patients were excluded. Eligibility criteria not met for 5 patients (patient under 75 years of age, *n* = 1; protected patient, *n* = 4) and iron workup was not done or done 48 h after inclusion for 9 patients. The analysis population included 888 patients.

Patients had a mean (SD) age of 85.2 (5.6) years and 63.5% were women (Table 1). Admission to the geriatric unit led to hospitalization in 52.1% of cases, and 47.9% of patients were seen on an outpatient basis. At least one comorbidity was reported in almost all patients (93.2%); the main comorbidities were arterial hypertension (67.5%), diabetes (21.5%), heart failure (21.3%), ischemic heart disease (19.5%), CKD (18.7%) and cancer (10.0%).

**Table 1:** Patient characteristics

Characteristics	N	Analysis population (*N* = 888)
Age, years, mean (SD)	888	85.2 (5.6)
Women, n (%)	888	564 (63.5)
Admission to geriatric unit, n (%)		
Hospitalized	888	463 (52.1)
Outpatients	888	425 (47.9)
Body mass index, kg/m^2^		
Mean (SD)	860	25.8 (5.1)
Classes, n (%)		
< 18.5 (underweight)	860	42 (4.9)
18.5–25 (normal)	860	374 (43.5)
25–30 (overweight)	860	278 (32.3)
≥ 30 (obesity)	860	166 (19.3)
≥ 1 comorbidity, n (%)	888	828 (93.2)
Arterial hypertension	888	599 (67.5)
Diabetes	888	191 (21.5)
Heart failure	888	189 (21.3)
Ischemic heart disease	888	173 (19.5)
Chronic kidney disease	888	166 (18.7)
Cancer	888	89 (10.0)
At least one treatment of ID or anemia, n (%)	888	142 (16.0)
Folic acid (ongoing at inclusion)	888	69 (7.8)
Vitamin B12 (ongoing at inclusion)	888	27 (3.0)
Oral iron (ongoing at inclusion)	888	39 (4.4)
Transfusion (within 3 months)	888	41 (4.6)
Intravenous iron (within 3 months)	888	23 (2.6)
ESA (within 3 months)	888	8 (0.9)
≥ 1 treatment with an impact on ID or anemia, n (%)	888	655 (73.8)
Anticoagulant	888	374 (42.1)
Proton pump inhibitor	888	303 (34.1)
Platelet antiaggregant	888	234 (26.4)
Other	888	28 (3.2)
Biological parameters, mean (SD)		
Serum ferritin, µg/L	872	262.5 (302.9)
Transferrin saturation (TSAT), %	852	23.9 (14.0)
Hb, g/dL	874	12.2 (1.9)
C-reactive protein, mg/L	869	24.4 (44.9)
Serum creatinine, µmol/L	879	92.6 (57.6)
Glomerular filtration rate,^a^ mL/min/1.73 m^2^	878	62.7 (20.1)
Serum albumin, g/L	853	
Mean (SD)		35.9 (6.1)
> 54		0
[34–54]^b^		560 (65.7)
< 34		293 (34.3)
Serum potassium, mmol/L	874	
Mean (SD)		4.13 (0.47)
> 5.5		5 (0.6)
[3.5–5.5]^b^		810 (92.7)
< 3.5		59 (6.8)

*ESA* erythropoiesis stimulating-agent, *Hb* hemoglobin, *ID* iron deficiency

^a^Calculated with CKD-EPI formula

^b^Normal range

Biological parameters are described in Table 1. Mean (SD) values of CRP were elevated at 24.4 (44.9) mg/L with CRP > 5 mg/L in 52.1% of patients.

At least one specific treatment of iron deficiency and/or anemia was reported by 16.0% of patients, either ongoing treatment (folic acid, 7.8%; Vitamin B12, 3.0%; oral iron, 4.4%) or within last 3 months (transfusion, 4.6%; intravenous iron, 2.6%; ESA, 0.9%) (Table 1).

At least one treatment with an impact on ID or anemia was reported at inclusion for 655 (73.8%) patients (anticoagulant, 42.1%; proton pump inhibitor, 34.1%; platelet antiaggregant, 26.4%).

### Prevalence of iron deficiency and/or anemia

The prevalence of ID was 57.6% (95% CI, 54.3–60.9) in the cohort of older patients admitted in geriatric unit (Table 2). Among ID patients, ID was absolute in 56.2% and functional in 43.8%.

**Table 2:** Prevalence of iron deficiency in analysis population

Parameters of iron deficiency	N	Analysis population (*N* = 888)
Iron deficiency^a^		
n (%)	859	495 (57.6)
95% CI, %	859	54.3–60.9
Absolute iron deficiency (serum ferritin < 100 µg/L), n (%)	495	278 (56.2)
Functional iron deficiency (serum ferritin ≥ 100 µg/L and TSAT < 20%), n (%)	495	217 (43.8)

*CI* confidence interval, *TSAT* transferrin saturation

^a^Defined as serum ferritin < 100 µg/L and/or TSAT < 20%

The prevalence of anemia was 48.5% (95% CI, 45.2–51.8). ID was significantly more frequent in anemic patients (62.6%; 95% CI 57.8–67.1) than in non-anemic patients (53.3%; 95% CI, 48.6–57.9; *p* = 0.0062).

**Fig. 1:**
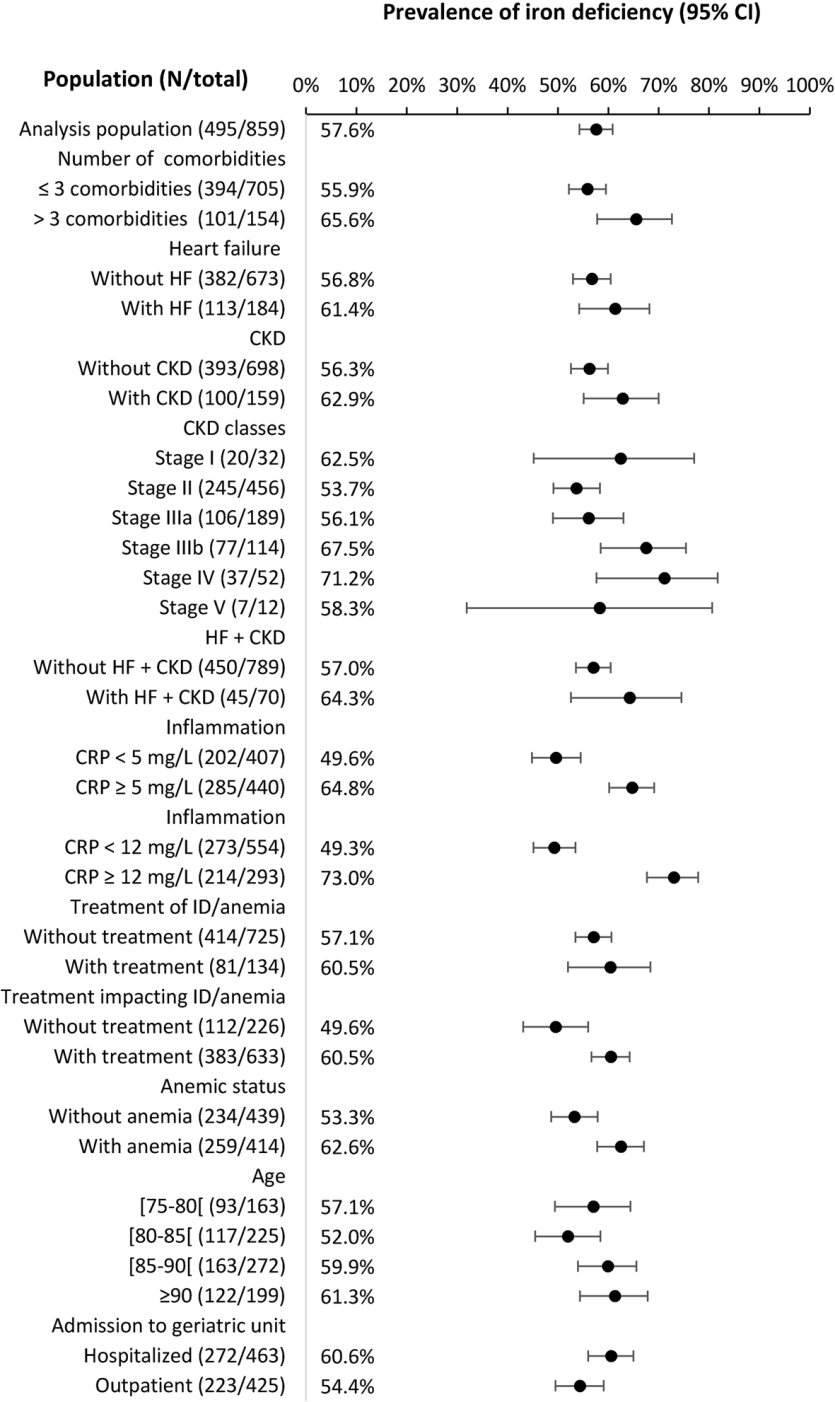
Prevalence of iron deficiency in different subgroups

ID was assessed in different subgroups presented in Fig. 1. ID was significantly more frequent in patients with > 3 comorbidities (65.6% vs. 55.9%; *p* = 0.0274), those receiving a treatment with a possible impact on ID or anemia (60.5% vs. 49.6%; *p* = 0.0042) or those with CRP ≥ 5 mg (64.8% vs. 49.6%; *p* < 0.001). The ROC curve method showed that a CRP threshold at 12 mg/mL was optimal for diagnosing ID. With this threshold, the difference of ID prevalence for inflammation vs. no inflammation was more marked (73.0% vs. 49.3%; *p* < 0.001). No specific pharmacological class for the treatment of ID/anemia or with a possible impact on ID could be evidenced with a significant effect on ID prevalence (Supplementary Table S1).

ID prevalence was high whatever patient age (from 52.0% to 61.3%) or the type of admission to geriatric unit (60.6% for hospitalized patients and 54.4% for outpatients) (Fig. 1). ID prevalence was 61.4% in patients with heart failure, 62.9% in patients with CKD and 64.3% patients with both CKD and heart failure.

In univariate analysis, > 3 comorbidities, CRP ≥ 12 mg/L, anemia, low serum albumin, and stages III-IV-V vs. I-II of renal failure were significantly associated with ID (Supplementary Table S2). In multivariate analysis, CRP ≥ 12 mg was the only independent predictive factor of ID (odds ratio, 2.78; 95% CI, 1.92–4.08; *p* < 0.001) (Supplementary Table S3).

### SPPB test scores in patients with iron deficiency

**Fig. 2:**
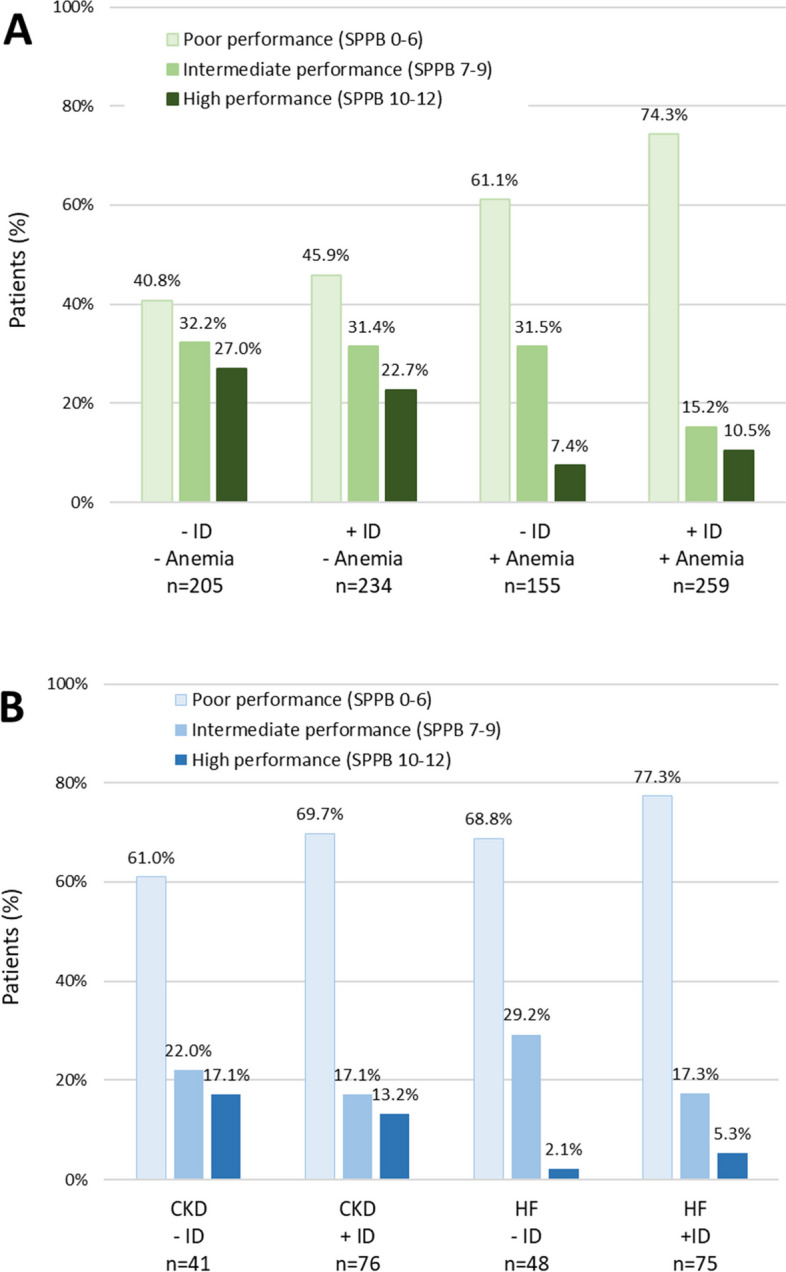
Physical performance (SPPB score) of older patients: **A**, according to iron deficiency (ID) and/or anemia; **B**, in patients with chronic kidney disease (CKD) or heart failure (HF)

In the 661 patients with available data for both iron status and SPPB test, scores were significantly lower in patients with ID than in patients without ID for global score and each dimension of SPPB (Table 3). Scores were low (SPPB 0–6) in 60.5% of patients with ID and in 48.6% of patients without ID (*p* = 0.0076). Low scores were reported in 40.8% of patients without ID and without anemia and in 74.3% of patients with both ID and anemia (Fig. 2A). In CKD patients, scores were low in 61.0% of patients without ID and 69.7% with ID; in patients with heart failure, these rates were 68.8% and 77.3%, respectively (Fig. 2B).

**Table 3:** SPPB test scores according to iron deficiency

SPPB items	N	Without ID *N* = 364	N	With ID *N* = 495	N	Total *N* = 859	*P*-value
Dimensions, median (IQR)							
Balance score	284	3 (1; 4)	377	2 (1; 4)	661	2 (1; 4)	0.0115
Gait speed score	284	3 (1; 4)	377	2 (1; 4)	661	2 (1; 4)	0.0445
Repeated chair stand score	284	1 (0; 3)	377	1 (0; 2)	661	1 (0; 2)	0.0101
Global score, median (IQR)	284	7 (4; 9)	377	5 (3; 9)	661	6 (3; 9)	0.0058
Global score interpretation, n (%)							
Poor performance (SPPB 0–6)	284	138 (48.6)	377	228 (60.5)	661	366 (55.4)	0.0076
Intermediate performance (SPPB 7–9)	284	91 (32.0)	377	87 (23.1)	661	178 (26.9)	
High performance (SPPB 10–12)	284	55 (19.4)	377	62 (16.4)	661	117 (17.7)	

*ID* iron deficiency, *IQR* interquartile range, *SPPB* Short Physical Performance Battery

## Discussion

In this large multicenter cross-sectional study, we found that more than half of patients admitted to a geriatric unit had ID (with about half absolute ID and half functional ID). Of note, half of ID patients were non-anemic. ID was associated with low SPPB score, which is predictive of high risk of adverse events (e.g., disability, falls, hospitalization, death).

Only few studies have specifically assessed the prevalence of ID in geriatric population while this topic is more often addressed in relation with anemia. As a consequence, ID-related anemia is often erroneously considered as a synonymous of ID. Because ID often precedes the onset of anemia, evaluating ID through only ID-related anemia underestimates the prevalence of ID. Thus, in the NHANES III study, nutrient deficiency was reported in about one-third of patients aged 65 years and older with anemia; half of these cases were related to ID [3]. In a single-center observational study performed in Germany in 2,191 patients ≥ 65 years undergoing major surgery, 791 were anemic (36.1%) and 276 (12.6%) were diagnosed with ID-related anemia [31]. In the DO-HEALTH study in 2,157 relatively healthy European adults aged 70 and older, ID prevalence defined by soluble transferrin receptor (sTfR) > 28.1 nmol/L was 26.8% [32]. In the study of Neidlein et al. in patients aged 65 years and over admitted to a geriatric ward, 41% had ID (defined as serum ferritin < 30 ng/mL and TSAT < 16% for absolute ID and serum ferritin ≥ 30 µg/L and TSAT < 16% for functional ID) [33]. The prevalence of non-anemic ID defined as serum ferritin < 30 µg/L was 8.8% in an English cohort of 4,451 adults of 45 years and older by Philip et al.; however this criterion evaluated only absolute ID [34]. Among 149 community-dwelling patients aged 55 years and older with a general good health status, ID (defined as serum ferritin < 15 µg/L or two other ID biomarkers) was diagnosed in 29.5% [35].

Comparisons between clinical studies are difficult because there is no consensus for definition of ID and for the thresholds of the available tests which frequently vary according to authors and clinical setting. For the present study, we have chosen a definition for which a consensus is emerging in patients with chronic diseases, namely serum ferritin < 100 μg/L and/or TSAT < 20% [25–27]. Indeed, in older patients, the underlying cause of ID is often multifactorial because several chronic comorbidities are frequently present. In the other CARENFER studies, high ID prevalence was reported in patients with cancer (57.9%) [6], heart failure (49.6%) [7], inflammatory bowel diseases (23.7%) [9] CKD (47.1%) [8] or preoperatively in patients undergoing major elective surgery (47.0%) [36]. By using a common definition of ID (serum ferritin < 100 μg/L and/or TSAT < 20%), the prevalence rates for the different chronic conditions were 58.1% in cancer, 62.8% in heart failure, 61.2% in inflammatory bowel disease and 47.1% in CKD [26]. A study in 101 US anemic veterans classified patients as iron deficient or iron sufficient on the basis of bone marrow hemosiderin, which is considered the gold standard for assessing iron stores [37]. Of interest, serum ferritin ≤ 100 µg/L had high specificity for the diagnosis of ID in these older patients with a wide variety of concomitant diseases and low TSAT had an even better diagnostic performance for ID. Other studies have also reported that the serum ferritin threshold for ID diagnosis should be higher for older patients than for the younger population [38, 39].

The patients from our cohort had the demographic characteristics and comorbidities of the geriatric population seen routinely in the hospital or included in real-life clinical studies [40]. Although the number of comorbidities aggravated ID, each of them had only a limited impact and ID seemed to be primarily associated with old person status. Only inflammation appeared to have a significant impact on ID prevalence and was the only independent factor predictive of ID in the multivariate analysis. Thus, at the CRP threshold of 12 mg/L, ID prevalence varied significantly from 49.3% (CRP < 12 mg/L) to 73.0% (CRP ≥ 12 mg/L). The inflammatory status is an important point to consider for ID treatment. Inflammation is often present in chronic diseases and has a significant impact on iron mobilization. Indeed, inflammation induces the synthesis of hepcidin by the liver; hepcidin in turn blocks iron export from intestinal cells and macrophages recycling iron, thus leading to a functional ID [2]. As a consequence, intravenous iron, which bypasses the intestinal barrier, is more effective than oral iron in inflammatory setting [41]. In our cohort, only a limited percentage of patients were treated for ID and/or anemia. Less than 3% received intravenous iron which is the preferred treatment of ID in inflammatory chronic diseases.

Patients with ID had lower scores to the SPPB test compared to patients without ID. Although we cannot conclude to a causal relationship between ID and low performance, these results are of interest since lower scores with SPPB have been associated with falls, hospitalization, long-term care needs, frailty and all-cause mortality [28]. In addition to erythropoiesis, iron is also involved in the physiology of skeletal muscle, which is highly dependent on iron for the synthesis of myoglobin and for energy production to support mechanical contraction [42]. Thus, ID is associated with impaired exercise capacity, due to a decrease of oxygen storage in myoglobin, decrease of energetic efficiency and mitochondrial dysfunction [43]. Our results are in line with those of Neidlein et al. who evaluated functional status (handgrip strength, isometric knee extension strength, walking, climbing stairs) during hospitalization in a geriatric population [33]. ID was an independent risk factor for fatigue and functional status on admission and during hospital stay.

The strengths of our study are the multicenter design and the large number of patients included. Our study is one of the few to have addressed the subject of ID in older patients, and such data were lacking in France. Moreover, our study is the first to evaluate physical performance in older patients with ID. However, our study has some limitations. Data were obtained from 16 centers spread over the French territory, but we cannot exclude some biases in patient recruitment, in particular because of the different profiles of hospitalized patients and outpatients. However, ID prevalence was high in each of the centers and therefore does not call into question the main conclusion of the study. The study was designed to evaluate ID prevalence and we did not evaluate the impact of iron supplementation, particularly on SPPB scores.

In conclusion, ID was diagnosed in more than half of patients admitted to a geriatric unit – both in non-anemic and anemic patients – and was associated to inflammation. ID was also associated to a low SPPB score which is a factor of poor prognosis. These new data underline the importance of more systematic screening of ID in the older population. Future randomized controlled trials will have to assess the efficacy of iron supplementation on physical performance and outcome of older iron-deficient patients.

### Supplementary Information

**Additional file 1: Supplementary Table S1. **Prevalence of iron deficiency according to treatment of iron deficiency/anemia or treatment with a possible impact on iron deficiency/anemia (analysis population, *N*=888). **Supplementary Table S2. **Univariate analysis of factors associated with iron deficiency (*N*=859). **Supplementary Table S3. **Multivariate analysis of factors associated with iron deficiency (*N*=859).

## Data Availability

The datasets used and analyzed during the current study will be made available from the corresponding author on reasonable request.

## Abbreviations

CKD: Chronic kidney disease
CRP: C-reactive protein
ESA: Erythropoiesis stimulating-agent
ID: Iron deficiency
SPPB: Short Physical Performance Battery
TSAT: Transferrin saturation coefficient
