# Supplementary material for: Prevalence of iron deficiency in patients admitted to a geriatric unit: a multicenter cross-sectional study
Source: BMC Geriatr. 2024 Jan 30;24:112. doi: 10.1186/s12877-024-04719-6 (PMC10826072; doi:10.1186/s12877-024-04719-6)
Supplement: Supplementary file 1 — Additional file 1: Supplementary Table S1. Prevalence of iron deficiency according to treatment of iron deficiency/anemia or treatment with a possible impact on iron deficiency/anemia (analysis population, N=888). Supplementary Table S2. Univariate analysis of factors associated with iron deficiency (N=859). Supplementary Table S3. Multivariate analysis of factors associated with iron deficiency (N=859). [file 12877_2024_4719_MOESM1_ESM.docx]

**Supplementary data**

**Supplementary Table S1.** Prevalence of iron deficiency according to treatment of iron deficiency/anemia or treatment with a possible impact on iron deficiency/anemia (analysis population, N=888).

| **Treatments** |  | **ID prevalence** | |
| --- | --- | --- | --- |
|  | **N** | **n (%)** | **95% CI** |
| **Treatment of iron deficiency/anemia** |  |  |  |
| Oral iron | 38 | 22 (57.9) | 42.2–72.1 |
| Intravenous iron | 23 | 11 (47.8) | 29.3–67.0 |
| Erythropoiesis-stimulating agent | 8 | 4 (50) | 21.7–78.3 |
| Folic acid | 66 | 39 (59.1) | 47.0–70.1 |
| Vitamin B12 | 24 | 14 (58.3) | 38.8–75.5 |
| Transfusion | 40 | 23 (57.5) | 42.2–71.5 |
| **Treatment with possible impact on iron deficiency** |  |  |  |
| Proton-pump inhibitor alone | 88 | 54 (61.4) | 50.9–70.8 |
| Anticoagulant alone | 207 | 119 (57.5) | 50.7–64.0 |
| Antiplatelet alone | 104 | 59 (56.7) | 47.1–65.8 |
| Proton-pump inhibitor plus anticoagulant | 108 | 68 (63.0) | 53.5–71.5 |
| Proton-pump inhibitor plus antiplatelet | 70 | 50 (71.4) | 59.9–80.7 |
| Anticoagulant plus antiplatelet | 26 | 15 (57.7) | 38.9–74.4 |
| Proton-pump inhibitor plus anticoagulant plus antiplatelet | 24 | 14 (58.3) | 38.8–75.5 |

**Supplementary Table S2.** Univariate analysis of factors associated with iron deficiency (N=859).

| **Variables** | | **Univariate analysis** | |
| --- | --- | --- | --- |
| **Factor** | **Comparison** | **Odds Ratio**  **(95% CI)** | **P-value** |
| Number of comorbidities | > 3 vs. ≤ 3 | 1.50 (1.05–2.18) | 0.0261 |
| Heart failure | Yes vs. No | 1.21 (0.87–1.70) | 0.2560 |
| Chronic kidney disease | Yes vs. No | 1.32 (0.93–1.88) | 0.1273 |
| Inflammation (CRP ≥ 12 mg/L) | Yes vs. No | 2.79 (2.06–3.81) | <0.0001 |
| Anemia | Yes vs. No | 1.46 (1.11–1.93) | 0.0062 |
| Serum albumin ^a^ | Abnormal vs. Normal | 1.50 (1.12–2.02) | 0.0063 |
| Serum potassium ^b^ | Abnormal vs. Normal | 1.18 (0.70–2.02) | 0.5485 |
| Stage of renal failure | Stages III-IV-V vs. I-II | 1.36 (1.04–1.80) | 0.0268 |

Factors with p-values ≤ 0.2 were selected for testing in an initial multivariate model before stepwise manual top-down selection with a risk alpha level set at p=0.05. CKD was not included because of its correlation with heart failure stage.

^a^ Normal serum albumin: [34–54] g/L; abnormal serum albumin: > 54 or < 34 g/L.

^b^ Normal serum potassium: [3.5–5.5] mmol/L: abnormal serum potassium: > 5.5 or 3.5 mmol/L.

**Supplementary Table S3.** Multivariate analysis of factors associated with iron deficiency (N=859).

| **Variables** | | **Multivariate analysis** | |
| --- | --- | --- | --- |
| **Factor** | **Comparison** | **Odds Ratio**  **(95% CI)** | **P-value** |
| Number of comorbidities | > 3 vs. ≤ 3 | 1.12 (0.74–1.71) | 0.5885 |
| Inflammation (CRP ≥ 12 mg/L) | Yes vs. No | 2.78 (1.92–4.08) | <0.0001 |
| Anemia | Yes vs. No | 1.14 (0.83–1.57) | 0.4270 |
| Serum albumin ^a^ | Abnormal vs. Normal | 0.78 (0.53–1.14) | 0.2027 |
| Stage of renal failure | Stages III-IV-V vs. I-II | 1.14 (0.84–1.56) | 0.3962 |

Multivariate logistic regression model modeling the association of factors with the probability of having iron deficiency. The stepwise descending factor selection method did not result in a multivariate model (i.e., with several factors significantly associated with ID).

^a^ Normal serum albumin: [34–54] g/L; abnormal serum albumin: > 54 or < 34 g/L.
